# Smart biomaterials: as active immune modulators to shape pro-regenerative microenvironments

**DOI:** 10.3389/fcell.2025.1669399

**Published:** 2025-10-14

**Authors:** Wenning Zhang, Xianyi Zeng, Xikai Deng, Fenglei Yang, Xiaoyuan Ma, Wei Gao

**Affiliations:** China Unicom Digital Intelligence Medical Technology Co. Ltd., Guangzhou, China

**Keywords:** smart biomaterials, macrophage polarization, immunomodulation, biomimetic materials, regenerative medicine

## Abstract

The field of smart biomaterials has evolved from passive scaffolds to dynamic, immune-modulating platforms capable of actively shaping regenerative microenvironments. This review explores the transition from inert to autonomous systems, emphasizing innovations in material responsiveness—such as pH, temperature, and enzymatic sensitivity—that enable intelligent interactions with biological cues. A key focus is the role of macrophage polarization in tissue repair, where biomaterials regulate immune responses through physicochemical properties and spatiotemporally controlled immunomodulatory factor release. Applications in cancer immunotherapy, myocardial regeneration, and scar inhibition highlight their therapeutic potential. Advances in biomimetic design and multiscale modeling accelerate rational development. However, clinical translation faces challenges in biosafety, scalability, and regulatory approval. Future directions point towards precision immune engineering, integrating optogenetic control, artificial intelligence-driven personalized design, and synergistic multimodal therapies. Ultimately, smart biomaterials are pioneering precision immune engineering, offering transformative strategies for regenerative medicine and disease intervention.

## 1 Introduction

The trajectory of biomaterials science has undergone a profound transformation, evolving from the development of passive constructs intended primarily for structural support to the engineering of sophisticated “smart” platforms. These advanced biomaterials are meticulously designed to actively and precisely interface with the host’s biological systems, particularly the immune system, to orchestrate desired therapeutic outcomes (Kowalski et al., 2018). This evolution is not merely an incremental improvement but represents a paradigm shift, fueled by the convergence of breakthroughs in materials science, immunology, and bioengineering. The capacity to intelligently interact with and guide cellular and tissue responses positions smart biomaterials at the forefront of regenerative medicine and advanced therapeutics.

The interaction between an implanted biomaterial and the host immune system is a critical determinant of its ultimate clinical success. An inappropriate or unresolved immune response can precipitate a cascade of adverse events, including chronic inflammation, the formation of a dense fibrous capsule that isolates the implant, and ultimately, implant failure and compromised tissue healing (Gomes et al., 2025). Recognizing this, the focus of biomaterial design has shifted from striving for mere “bio-inertness”—a state of passive coexistence—to achieving active “immunomodulation”. Smart biomaterials are engineered to engage with the immune system, particularly innate immune cells such as macrophages, and to skillfully guide their behavior towards a pro-regenerative, anti-inflammatory, or anti-fibrotic phenotype (Raheem et al., 2025). This strategic manipulation aims to create a local microenvironment that is conducive to constructive tissue repair and functional restoration, rather than destructive inflammation or scarring. This conceptual pivot acknowledges the immune system not as an adversary to be evaded, but as a powerful biological system that can be rationally programmed and harnessed for therapeutic benefit.

At the heart of this new generation of biomaterials lie two core innovative principles: “dynamic responsiveness” and “active regulatory capabilities”. The “smartness” is embodied in their ability to sense and react to specific physiological or pathological cues within their local microenvironment, such as alterations in pH, temperature fluctuations, the presence of specific enzymes, or changes in redox status. In response to these stimuli, these materials can dynamically alter their own physicochemical properties—for instance, changing their stiffness, swelling or shrinking, or modifying their surface chemistry. Alternatively, or concurrently, they can execute programmed actions, such as the precisely controlled release of encapsulated immunomodulatory factors (Hotaling et al., 2015). This responsiveness is not a simple on-off switch; rather, it allows for interventions that are both spatially confined to the target tissue and temporally orchestrated to match the dynamic phases of biological processes like wound healing or immune response. Such capabilities effectively transform the biomaterial from a static implant into an *in situ* therapeutic delivery and control system, blurring the traditional distinctions between a medical device and a pharmacological agent. This integration offers a more holistic and adaptive treatment modality compared to the separate administration of passive scaffolds and systemically delivered drugs, posing new considerations for regulatory frameworks and the fundamental definition of medical interventions.

The capacity to sculpt pro-regenerative microenvironments through such sophisticated immune modulation opens up a vast spectrum of therapeutic applications across diverse medical disciplines. The principles underpinning the immunomodulatory actions of smart biomaterials appear to be broadly applicable, targeting common cellular and molecular pathways, particularly macrophage polarization and cytokine networks, irrespective of the specific tissue or disease context. This commonality facilitates a valuable cross-pollination of technologies and insights across fields. Consequently, smart biomaterials are making significant inroads in orthopedics, for applications such as promoting bone regeneration and developing intelligent implants; in dentistry, for enhancing dental pulp repair and improving the osseointegration of dental implants; in managing cardiovascular diseases, through strategies for myocardial infarction repair and the creation of advanced vascular grafts; and in oncology, by developing novel approaches to enhance cancer immunotherapy and modulate the tumor microenvironment (Kowalski et al., 2018).

This review aims to provide a comprehensive exploration of the design principles, underlying mechanisms of action, and diverse therapeutic applications of smart biomaterials as active immune modulators. It will delve into their remarkable capacity to orchestrate pro-regenerative microenvironments, critically examine the current challenges impeding their widespread clinical translation, and discuss the future innovations and perspectives that are poised to further revolutionize the field of regenerative medicine and precision immune engineering.

## 2 Definition and classification of smart biomaterials

The “intelligence” ascribed to smart biomaterials is fundamentally rooted in their engineered capacity to sense specific alterations in their surrounding environment and to respond to these changes in a predetermined, functional manner. These environmental triggers can be endogenous, arising from physiological or pathological processes within the host, such as shifts in pH, temperature variations, the presence of specific enzymatic activities, fluctuations in glucose concentrations, or changes in the local redox state. Alternatively, stimuli can be exogenous, applied externally, such as light, magnetic fields, or ultrasound. Upon sensing such stimuli, smart biomaterials can dynamically adjust their physicochemical characteristics—for example, altering their stiffness, undergoing conformational changes, or modifying their surface hydrophilicity—or they can initiate the controlled release of encapsulated bioactive agents with precise spatial and temporal control (Narkar et al., 2022). A significant conceptual leap in this field is the evolution of the definition beyond simple stimulus-response to encompass “bi-directional responsiveness”. This advanced concept envisions materials that not only influence cellular activity but are also capable of receiving feedback signals from the cells and remodeling themselves accordingly, thereby creating truly adaptive and interactive systems.

**FIGURE 1:**
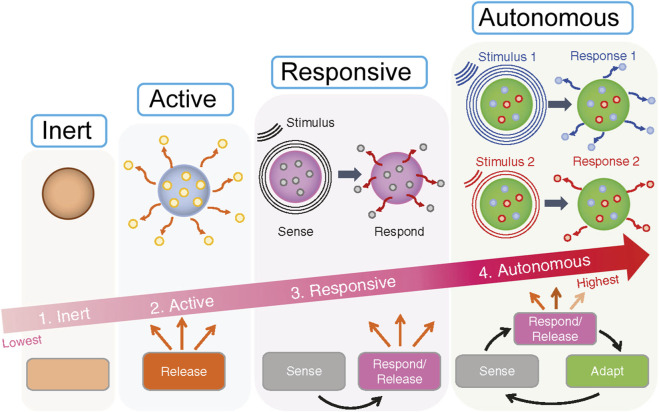
The four levels of smartness for biomaterials: (1) Inert, (2) Active, (3) Responsive, (4) Autonomous. It illustrates progression from inert materials which do nothing, to active releasing materials, responsive ones that sense and respond, culminating in autonomous materials that sense, respond, release, and adapt to stimuli. An arrow indicates increasing smartness from inert to autonomous.

The classification of biomaterials often reflects an increasing level of sophistication in their interaction with biological systems, essentially mirroring an evolution in their biomimetic capabilities (Figure 1). Where inert materials are largely non-biomimetic in their function, merely occupying space, active materials begin to emulate simple biological actions. Responsive materials mimic conditional biological processes, and autonomous materials aim to replicate complex biological control systems, such as homeostatic feedback loops. This progression highlights a journey from static implants to dynamic, self-adaptive platforms (Montoya et al., 2021).

### 2.1 Inert materials

These materials are designed primarily for structural support or to occupy a void, with an emphasis on eliciting minimal biological interaction and toxicity. They do not induce a specific, intended biological response beyond the normal wound healing cascade. Traditional examples include certain grades of titanium alloys, some ceramics, and various inert polymers (Raheem et al., 2025). A primary limitation of inert materials is their propensity to trigger a foreign body response (FBR), often culminating in the formation of a fibrous capsule that isolates the implant and can compromise its function.

### 2.2 Active materials (or bioactive materials)

This category represents a step beyond inertness, encompassing materials designed to elicit a defined biological response at the material-tissue interface. This can be achieved through the release of pre-loaded bioactive agents, such as drugs, growth factors, or anti-inflammatory molecules, or via inherent surface bioactivity that promotes favorable cell adhesion, tissue integration, and specific cellular functions (Hotaling et al., 2015). Examples include drug-eluting stents that release anti-proliferative agents, antibiotic-loaded bone cements, and hydroxyapatite coatings on orthopedic implants that enhance osseointegration. The mechanisms are typically based on passive diffusion of loaded agents or direct surface-mediated cellular interactions.

### 2.3 Responsive materials

#### 2.3.1 Stimuli-responsive materials

These materials are engineered to undergo a significant change in their properties or to trigger a specific action, such as drug release or a structural transformation, upon encountering a specific stimulus. The stimuli are often direct indicators of pathological or regenerative states, allowing the material to function almost as a localized “diagnostic-therapeutic” (theranostic) entity. Chen et al. reported a three functional stimulus responsive nanosystem based on superparamagnetic Fe_3_O_4_ and paramagnetic MnOx nanoparticles (NPs) co integrated onto exfoliated graphene oxide (GO) nanosheets using a novel and efficient double redox strategy (DRS). Aromatic anticancer drug molecules can interact with GO nanosheets through supramolecular π stacking, achieving high drug loading and pH responsive drug release performance (Chen et al., 2014).

#### 2.3.2 pH-responsive materials

These materials exploit the acidic microenvironments often found in tumors (pH 6.5–6.9) or sites of inflammation. They may contain ionizable groups that lead to swelling, de-swelling, or conformational changes, or incorporate pH-labile chemical bonds (e.g., hydrazones, acetals, orthoesters) that cleave to release an encapsulated payload (Narkar et al., 2022).

#### 2.3.3 Temperature-responsive materials

Polymers such as poly(N-isopropylacrylamide) (PNIPAM) are well-known examples that exhibit a lower critical solution temperature (LCST). Below the LCST, they are typically soluble and hydrated; above it, they become hydrophobic and can precipitate or form a gel. This sol-gel transition can be used for injectable systems that solidify *in situ* at body temperature or for modulating drug release.

#### 2.3.4 Enzyme-responsive materials

These materials are designed with components that are specifically recognized and cleaved by enzymes overexpressed in certain pathological conditions, such as matrix metalloproteinases (MMPs) in chronic wounds, inflammatory sites, or tumor tissues. Enzymatic action leads to material degradation or payload release. Yang et al. reported that a multi-component enzyme reactive natural polymer, hyaluronic acid (HA) microneedles, was embedded in cerium/zinc-based nanomaterials (ZCO) for the treatment of diabetes wounds. ZCO-HA can disrupt the oxidative balance of bacteria, kill them, eliminate reactive oxygen species (ROS), and alleviate oxidative stress by regulating the release of Zn^2+^. *In vivo* studies on streptozotocin induced diabetes mice showed that this microneedle could accelerate wound healing without systemic toxicity (Yang et al., 2023).

#### 2.3.5 Light-responsive materials

Incorporation of photo-sensitive moieties (e.g., azobenzene, spiropyran) allows these materials to change their properties (e.g., conformation, hydrophilicity, drug release) upon exposure to specific wavelengths of light, offering external spatiotemporal control (Raheem et al., 2025). Other stimuli include redox potential, specific analytes like glucose, magnetic fields, and ultrasound.

### 2.4 Autonomous materials (self-adaptive/bi-directional materials)

This represents the most advanced and aspirational class of smart biomaterials. These systems aim to integrate sensing elements, processing logic (potentially rudimentary), and actuating components to autonomously adapt their behavior in real-time response to the dynamic local microenvironment. They embody the concept of a closed-loop control system. Examples, though many are still conceptual or in early development, include self-healing polymers that can repair damage, materials with integrated biosensors that detect specific biomarkers and trigger a therapeutic response, and the envisioned “bi-directional ECM platforms” capable of dynamic, reciprocal communication with cells. “Self-aware implants” that can measure physiological parameters, record data, transmit it wirelessly, and potentially self-power also fall under this umbrella. The development of such materials necessitates a convergence of materials science with principles from information technology and control systems, potentially leading to “cybernetic” biomaterials that can execute complex, pre-programmed biological interventions or maintain local homeostasis.

This evolutionary trajectory from static to self-adaptive materials underscores a continuous effort to create biomaterials that not only coexist with biological systems but can intelligently and beneficially interact with them, moving ever closer to mimicking the sophisticated regulatory mechanisms found in nature. The ultimate goal is to develop materials that can faithfully recreate the dynamic and instructive nature of the native cellular microenvironment. Table 1 provides a structured overview of these classifications, highlighting their core definitions, key response mechanisms, levels of “intelligence” and representative examples.

**TABLE 1:** Classification and characteristics of smart biomaterials.

Class	Core definition	Key stimuli/ Response mechanisms	Level of “intelligence”/Interaction	Representative examples
Inert Materials	Provide structural support or fill space with minimal intended biological interaction or toxicity.	(a) Primarily mechanical support. (b) No specific stimulus-response.	Passive	Traditional titanium alloys, certain ceramics (e.g., alumina, zirconia) (Raheem et al., 2025)
Active Materials	Elicit a specific biological response via pre-loaded agents or inherent surface properties.	(a) Pre-loaded drug/growth factor release (often diffusion-controlled). (b) Surface chemistry promoting cell adhesion.	Pre-programmed release; Interaction	Drug-eluting stents, antibiotic-loaded bone cements, hydroxyapatite coatings (Hotaling et al., 2015)
Responsive Materials	Undergo changes in properties or trigger actions in response to specific internal/external stimuli.	(a) pH: Swelling/de-swelling, degradation of labile bonds, charge alteration leading to drug release (e.g., in acidic tumor/inflammatory sites). (b) Temperature: Sol-gel transition (e.g., PNIPAM), conformational changes affecting drug release. (c) Enzymes: Degradation of material by specific enzymes (e.g., MMPs, hyaluronidase) triggering payload release. (d) Light: Conformational change, cleavage of photosensitive linkers, photothermal effect leading to release. (e) Redox: Cleavage of disulfide/diselenide bonds, oxidation/reduction of responsive moieties.	Conditional response	(a) pH-sensitive hydrogels/nanoparticles for tumor drug delivery (Yang et al., 2025a). (b) Temperature-sensitive polymers (e.g., PNIPAM-based) for injectable scaffolds/drug delivery (Narkar et al., 2022). (c) Enzyme-responsive scaffolds (e.g., MMP-sensitive) for wound healing/cancer. (d) Light-responsive materials for controlled release.
Autonomous Materials	Integrate sensing, processing, and actuation for real-time adaptation to microenvironmental changes; may involve feedback loops.	(a) Integrated sensors detect biomarkers/physiological changes. (b) Feedback systems trigger adaptive responses. (e.g., adjust drug release, modify material properties). (c) Bi-directional communication with cells leading to mutual adaptation.	Adaptive feedback control; Bi-directional communication	(a) Conceptual: Self-healing polymers, materials with integrated biosensor-actuator systems, bi-directional ECM platforms (Narkar et al., 2022); (b) Self-aware implants with data transmission and self-powering capabilities (Raheem et al., 2025).

## 3 Active regulation of immune cell behavior via physicochemical properties

Biomaterials implanted into living tissue are not passive entities; they engage in a dynamic dialogue with host cells, mediated by a complex array of physical and chemical signals. Immune cells, with macrophages at the forefront, are particularly attuned to the physicochemical characteristics of their surroundings (Raheem et al., 2025). Properties such as substrate stiffness (mechanics), surface topography (micro- and nanoscale architecture), and the rate and nature of material degradation (which dictates the release of chemical cues and alters the physical landscape) serve as potent, non-pharmacological regulators of immune cell phenotype and function. This understanding forms the basis for designing “immuno-instructive” biomaterials that can actively guide immune responses towards regeneration.

### 3.1 Stiffness-mediated macrophage polarization

**FIGURE 2:**
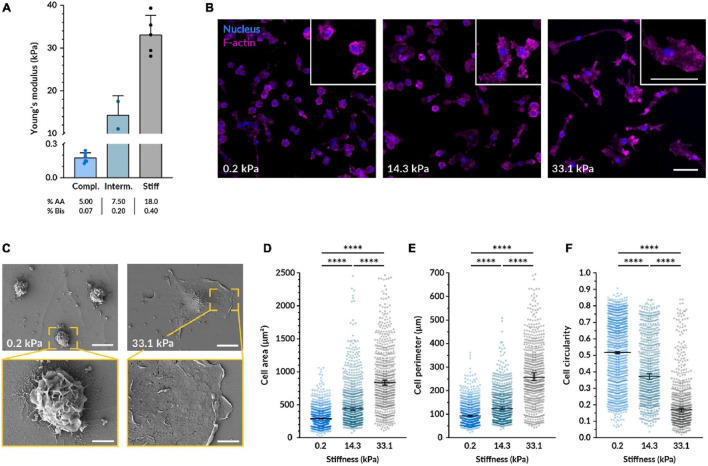
Substrate stiffness affects the morphology of macrophages. **(A)** Bar graph showing Young's modulus for compliant, intermediate, and stiff substrates with increasing values. **(B)** Fluorescence images of cells on substrates with varying stiffness (0.2, 14.3, and 33.1 kPa) highlighting nuclei in blue and F-actin in pink. **(C)** SEM images displaying cells on 0.2 and 33.1 kPa substrates with zoomed insets showing detailed cell morphology. **(D–F)** Quantification of the cell area **(D)**, perimeter **(E)**, and circularity **(F)** from images taken under the same conditions as **(B)**.

The mechanical stiffness, or elasticity, of a biomaterial substrate has emerged as a critical determinant of macrophage behavior, capable of profoundly influencing their polarization towards either a pro-inflammatory (M1-like) or an anti-inflammatory/pro-reparative (M2-like) state. This phenomenon suggests that macrophages can “feel” their environment and adjust their functional programming accordingly. Softer substrates, often defined in the range of <10 kPa (though specific effective ranges can vary depending on the material system and cell type), are frequently reported to encourage M2-like polarization. This effect may be mediated through the inhibition of key pro-inflammatory signaling pathways, such as the NF-κB pathway (Escolano et al., 2021). Conversely, stiffer substrates (e.g., >100 kPa or simply reflecting a more rigid environment) have been shown to promote M1-like polarization. This can occur through the activation of mechanosensitive structures like the NLRP3 inflammasome, which is a key component of the innate immune response, or by fostering increased actomyosin contractility within the macrophage. For example, studies have demonstrated that compliant substrates can enhance inflammasome formation and the release of pro-inflammatory cytokines like IL-1β and IL-6, while others link stiff substrates to NLRP3 activation (Figure 2).

**FIGURE 3:**
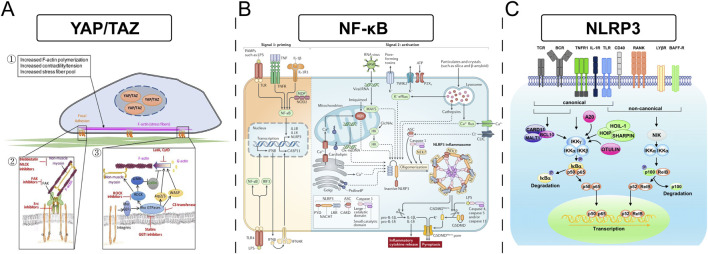
**(A)** The YAP/TAZ signaling pathway involved in increased F-actin polymerization, contractility, and stress fiber formation. **(B)** The NF-κB pathway, highlighting TLR and NLRP3 inflammasomes in immune response activation. **(C)** Describes canonical and non-canonical NLRP3 pathways, showing components like CARD11 and OTULIN affecting NF-kB-mediated transcription.

It is important to note that the relationship between stiffness and macrophage polarization is not always linear; a simple “soft equals M2, stiff equals M1” paradigm may be an oversimplification. Some investigations indicate that compliant substrates can, under certain conditions, also enhance pro-inflammatory responses, suggesting a more complex, possibly biphasic or optimal range-dependent, cellular interpretation of mechanical cues. Discrepancies in the literature likely arise from variations in experimental setups, including the specific cell lines or primary macrophages used, the density and type of adhesion proteins coating the substrates, the nature of co-stimulatory signals present, and the specific stiffness ranges investigated. Key signaling pathways implicated in macrophage mechanosensing include NF-κB, the NLRP3 inflammasome, and pathways regulating cytoskeletal dynamics and contractility, such as Rho/ROCK signaling. The Yes-associated protein (YAP) and transcriptional co-activator with PDZ-binding motif (TAZ), well-known mediators of mechanotransduction in other cell types, are also increasingly recognized for their potential role in macrophage responses to stiffness. The material’s physical properties are not just passive background features but are actively “interpreted” by cells through intricate mechanotransduction pathways, leading to the activation of specific gene expression programs. This implies the existence of a “mechanical language” that biomaterials can leverage to communicate with and instruct immune cells (Figure 3).

### 3.2 Topology-guided immune cell migration and function

The surface topography of a biomaterial—encompassing features such as nanoscale grooves, fibers, pores, pillars, and sheets—can profoundly influence immune cell behavior by mimicking aspects of the natural extracellular matrix (ECM). These topographical cues can direct cell adhesion, morphology, migration patterns, and cytokine secretion profiles. Biomimetic nanoscale grooves or fibers, often with diameters or feature sizes in the range of 50–500 nm (e.g., 400–500 nm wide grooves for macrophage elongation, or the 50–200 nm range mentioned in the user query), can induce contact guidance, leading to aligned cell migration and altered cell shape. For macrophages, an elongated morphology is frequently associated with an M2-like, pro-repair phenotype (Liu et al., 2023).

Nanotopography exerts its influence by modulating the formation of focal adhesions and orchestrating the rearrangement of the cell’s cytoskeleton, particularly actin filaments and microtubules. These cytoskeletal changes are integral to cell motility and the transduction of mechanical signals into biochemical responses. Such physical cues can translate into altered gene expression, leading, for example, to enhanced secretion of anti-inflammatory cytokines like IL-10. Studies have shown that Mg-Al layered double hydroxide nano-sheet arrays on titanium surfaces promoted M2 macrophage polarization and IL-10 secretion, an effect linked to the activation of PI3K-AKT-mTOR signaling and increased expression of integrin β2 and focal adhesion kinase (FAK) (Zheng et al., 2022). Similarly, biomimetic ECM nerve guidance conduits featuring a 3D interconnected porous network have been shown to promote M2 polarization (Wan et al., 2025). The specificity of these responses to varied topographies suggests a high degree of sophistication in cellular mechanosensing, implying that achieving a desired immunomodulatory outcome often requires multi-parametric optimization of biomaterial design rather than focusing on a single physicochemical property.

### 3.3 Degradation rate and fibrosis balance

The rate at which a biomaterial degrades is a critical parameter that influences the local immune response and the ultimate fate of an implant (Mariani et al., 2019). Degradation kinetics determine the duration of the material’s presence, the profile of released byproducts, and the dynamic changes in the material’s physical properties, all of which significantly impact macrophage phenotype switching and the delicate balance between constructive tissue repair and detrimental fibrosis. Rapidly degrading materials may release a sudden bolus of byproducts, potentially overwhelming local clearance mechanisms and triggering an acute, intense M1-dominant inflammatory response. If this inflammation is not resolved efficiently, it can transition into a chronic state, fostering fibrosis. Acidic degradation products, such as those from poly(lactic-co-glycolic acid) (PLGA), can lower the local pH, which can further modulate cell behavior and, if excessive, cause localized tissue damage or necrosis (Mariani et al., 2019).

In contrast, a controlled, slower degradation profile can facilitate a more coordinated and beneficial immune response. It allows for an initial M1 phase, necessary for clearing debris and pathogens, followed by a timely transition to an M2-dominant phase, which supports tissue remodeling, angiogenesis, and organized ECM deposition, thereby minimizing excessive collagen accumulation and promoting functional tissue regeneration (Li et al., 2024). The dynamic changes in surface properties of a degrading material also contribute to modulating cellular interactions over time (Mariani et al., 2019). Furthermore, the size and shape of the degradation particles can influence macrophage responses, for instance, larger particles might be less readily phagocytosed and could bias towards M2 phenotypes, whereas smaller particles might elicit different activation states. PLGA is a commonly studied biodegradable polymer whose degradation rate can be tailored by adjusting its monomer ratio (lactic acid to glycolic acid) and molecular weight. While generally considered biocompatible, the management of its acidic byproducts is a key design consideration. The interplay between degradation rate and macrophage phenotype highlights a crucial temporal dimension in immunomodulation. Successful regeneration often requires not just achieving an M2 state, but achieving it at the appropriate time, synchronized with other phases of healing. This points towards the necessity for 4D biomaterials, where material properties, including degradation and subsequent signaling, change predictably over time to actively guide the sequential stages of the immune response.

These intricate relationships between material stiffness, topography, and degradation kinetics provide a robust theoretical basis for the rational design of “immune-friendly” or, more accurately, “immuno-instructive” biomaterials. The objective is not necessarily to create materials that are completely “stealthy” and evade all immune detection, but rather to engage the immune system in a controlled, predictable, and ultimately beneficial manner, steering it towards a pro-regenerative trajectory. Table 2 summarizes the impact of key biomaterial physicochemical properties on macrophage polarization and function, drawing from the discussed mechanisms and examples.

**TABLE 2:** Impact of biomaterial physicochemical properties on macrophage polarization and function.

Physicochemical property		Predominant macrophage phenotype induced	Key immunomodulatory outcomes	Associated signaling pathways (if identified)	Example biomaterials & citation
Substrate Stiffness	Soft (<10 kPa)	Often M2-like, but highly context-dependent.	↓Pro-inflammatory cytokines, ↑Anti-inflammatory cytokines, reduced fibrosis	Inhibition of NF-κB	Polyacrylamide hydrogels (Escolano et al., 2021)
	Stiff (>100 kPa)	Often M1-like	↑Pro-inflammatory cytokines, potential for increased fibrosis if prolonged	Activation of NLRP3 inflammasome, ↑Actomyosin contractility, Rho/ROCK	Polyacrylamide hydrogels (Escolano et al., 2021)
Surface Topography	Nanoscale grooves (e.g., 400-500nm width)	M2-like (elongated morphology)	↑Anti-inflammatory markers (e.g., Arg-1), guided migration	Cytoskeletal rearrangement	PCL, PDMS, PLA with grooves (Liu et al., 2023)
	Nano-sheet arrays (Mg-Al LDH on Ti)	M2-like	↑IL-10, ↑Arg-1, promotion of osteogenesis via mBMSCs	PI3K-AKT-mTOR, Integrin β2, FAK	Mg-Al LDH on titanium (Zheng et al., 2022)
	Micropillars (e.g., 5-10 µm diameter, dense)	M2-like	Enhanced attachment, promotion of anti-inflammatory phenotype		Various polymers (Liu et al., 2023)
	Honeycomb TiO2 nanostructures	M2-like	↑Anti-inflammatory cytokines	Rho family protein signaling	TiO2 surfaces (Lamers et al., 2012)
Degradation Profile	Rapid degradation with acidic byproducts	Initially M1-dominant (acute inflammation)	↑Pro-inflammatory cytokines, risk of chronic inflammation and fibrosis if unresolved, potential tissue damage from acidity	Inflammatory signaling	Rapidly degrading PLGA (Mariani et al., 2019)
	Controlled, slow degradation	Coordinated M1→ M2 transition	Initial M1 for clearance, then sustained M2 for repair, reduced fibrosis, organized tissue regeneration	Balanced inflammatory and repair signaling	Tuned PLGA, other biodegradable polymers (Li et al., 2024)

## 4 Spatiotemporal controlled release strategies for immunomodulatory factors

The systemic administration of potent immunomodulatory agents, such as cytokines, small-molecule drugs, and nucleic acids, is frequently hampered by significant challenges, including dose-limiting systemic toxicities and undesirable off-target effects (Nash et al., 2021). Smart biomaterial-based carrier systems offer a compelling solution to these limitations by enabling the localized delivery and precise spatiotemporal control over the release of these therapeutic factors. This targeted approach aims to maximize therapeutic efficacy directly at the site of injury or disease while concurrently minimizing systemic exposure and associated adverse events (Yang et al., 2025a). The evolution of these delivery systems reflects an increasing biomimicry of natural biological transport and signaling mechanisms, with engineered extracellular vesicles (EVs), for example, leveraging endogenous cellular communication pathways.

### 4.1 Responsive nanocarriers

Responsive nanocarriers are nanoparticles engineered to encapsulate therapeutic payloads and release them selectively in response to specific triggers present in the target microenvironment. This “on-demand” release enhances therapeutic precision.

#### 4.1.1 pH-responsive nanoparticles

These systems are designed to exploit the acidic conditions often found in pathological tissues, such as the tumor microenvironment (TME, pH typically 5.5–6.8) or sites of inflammation (Karimi et al., 2025). The acidic pH can trigger drug release through various mechanisms, including the cleavage of pH-labile chemical linkers (e.g., hydrazones, acetals, ketals), the protonation of ionizable polymer groups leading to swelling or disassembly of the nanoparticle, or the dissolution of acid-sensitive inorganic components (Yang et al., 2025a). A key application is in cancer immunotherapy, where pH-responsive nanoparticles can deliver immunostimulatory agents like Interleukin-12 (IL-12) directly within the TME to activate T cells and enhance anti-tumor responses, or release immune checkpoint inhibitors such as anti-PD-L1 antibodies (Liu et al., 2025). For instance, pH-responsive nano-vaccines have been developed to release STING agonists and neoantigens in acidic conditions, promoting dendritic cell activation and T-cell responses, which can be synergistic with systemic anti-PD-1 therapy (Wang et al., 2025a).

#### 4.1.2 Redox-responsive nanoparticles

These carriers are sensitive to the distinct redox potentials found in different cellular compartments or between normal and pathological tissues. For example, the intracellular environment, particularly in tumor cells, often has a higher concentration of reducing agents like glutathione (GSH) compared to the extracellular space. Conversely, sites of inflammation can be rich in reactive oxygen species (ROS). Redox-responsive systems commonly incorporate disulfide bonds, which are cleaved by GSH, or employ polymers containing selenium or tellurium, which are susceptible to oxidation by ROS, leading to nanoparticle disassembly and drug release. Applications include the delivery of anti-cancer drugs to tumor cells or the release of anti-inflammatory agents and ROS scavengers in inflamed tissues (Yang et al., 2025a).

#### 4.1.3 Enzyme-responsive nanoparticles

These systems are tailored to respond to enzymes that are specifically overexpressed or hyperactive in diseased tissues. For example, matrix metalloproteinases (MMPs) are often upregulated in tumors, chronic wounds, and arthritic joints, while phospholipase A2 (PLA2) may be active in wound exudates.^11^ Nanoparticles can be constructed with enzyme-cleavable linkers or components, such that enzymatic activity at the target site triggers payload release. This strategy allows for highly specific drug delivery, for instance, releasing anti-inflammatory drugs in MMP-rich arthritic joints or anti-cancer agents in the tumor microenvironment.

### 4.2 Multi-stage release systems

Beyond single-stimulus responsiveness, more sophisticated systems are being developed for multi-stage release. These platforms are designed to deliver multiple therapeutic agents in a programmed sequence or to release a single agent with complex, temporally defined kinetics (e.g., an initial burst followed by sustained release, or pulsatile delivery). Such temporal control is crucial for therapies that require the sequential action of different biological signals. For instance, in the context of autoimmune diseases, biomaterials can be engineered for the sequential delivery of IL-2, to promote the expansion and survival of regulatory T cells (Tregs), followed by TGF-β1, to enhance their suppressive function or induce *de novo* Treg differentiation. Studies have demonstrated the release of IL-2, TGF-β, and rapamycin from microparticle formulations over several weeks, successfully inducing functional Tregs *in vitro* (Jhunjhunwala et al., 2012). This approach reflects a sophisticated understanding that effective immunomodulation often involves orchestrating a precise sequence of signals to guide complex cellular pathways, rather than a simple “on” or “off” activation.

### 4.3 Engineered extracellular vesicles (EVs)

Extracellular vesicles, including exosomes (typically 30–150 nm) and micro-vesicles, are naturally secreted, membrane-bound nanoparticles that play a crucial role in intercellular communication by transporting a diverse cargo of proteins, lipids, and nucleic acids (mRNAs, miRNAs) between cells (Wang et al., 2025b). Their inherent biocompatibility, low immunogenicity, and natural ability to traverse biological barriers make them attractive candidates for drug delivery (Abudurexiti et al., 2023). EV engineering involves modifying these natural carriers to enhance their therapeutic potential. This can be achieved through:

**Endogenous modification:** Genetically altering the parent cells to produce EVs enriched with specific therapeutic molecules.

**Exogenous modification:** Directly loading therapeutic cargo into isolated EVs using techniques such as electroporation, sonication, extrusion, or simple co-incubation.

**Surface display:** Engineering the EV surface to present targeting ligands for specific cell types or to display immunomodulatory proteins. This can be done by fusing the protein of interest to EV-associated proteins like Lamp2b or CD63.

A compelling application of engineered EVs is the display of co-stimulatory molecules (e.g., CD80, OX40L) or co-inhibitory molecules (e.g., PD-L1) on their surface. Such “immunologically active” EVs can directly engage T-cell receptors and bidirectionally regulate T-cell activity, offering novel strategies for cancer immunotherapy or the treatment of autoimmune diseases (Kugeratski et al., 2023). For example, EVs engineered to express murine OX40L (mOX40L EVs) demonstrated significant tumor growth delay in preclinical models by promoting CD8^+^ T cell infiltration and enhancing their cytotoxic potential. Conversely, mPD-L1 EVs accelerated tumor growth by suppressing T cell activity. This approach effectively transforms EVs from passive delivery vehicles into active immunotherapeutic agents, blurring the lines between drug delivery and cell-based therapies and potentially offering some benefits of cell therapy without the complexities of administering live cells. EVs are also being explored for the delivery of regulatory miRNAs, such as miRNA-223, to modulate macrophage polarization (Saadh et al., 2025).

The overarching advantage of these smart carrier strategies—responsive nanocarriers, multi-stage systems, and engineered EVs—lies in their capacity to achieve high local concentrations of therapeutics at the desired site of action while maintaining low systemic levels. This spatiotemporal control significantly broadens the therapeutic window for potent immunomodulators, thereby reducing dose-limiting toxicities and enhancing both the safety and efficacy of treatment regimens (Yang et al., 2025a).

## 5 Macrophage polarization: The pivotal hub between tissue repair and fibrosis

**FIGURE 4:**
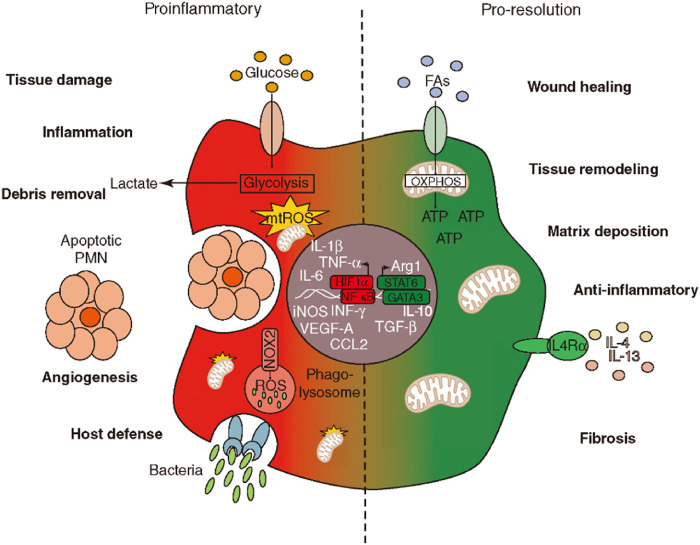
The balance between proinflammatory and pro-resolution processes. The left side illustrates proinflammatory responses with elements like glucose intake, glycolysis, and debris removal. It highlights factors such as IL-1ß and TNF-α. The right side represents pro-resolution processes, focusing on wound healing, tissue remodeling, and anti-inflammatory responses, with elements like IL-4 and IL-13. The transition between these states involves metabolic pathways, including OXPHOS for ATP production and LC4α receptor activation. The diagram showcases molecular and cellular components involved in inflammation and resolution phases.

Macrophages, as versatile cells of the innate immune system, exhibit remarkable plasticity and heterogeneity, enabling them to adopt a wide spectrum of activation states in direct response to the complex array of cues present in their microenvironment (Willenborg et al., 2022). While the classical M1 (pro-inflammatory) and M2 (anti-inflammatory/pro-reparative) dichotomy provides a useful framework for understanding macrophage functions, it is increasingly recognized that macrophage activation represents a continuum rather than two discrete states. Advanced techniques like single-cell RNA sequencing (scRNA-seq) are progressively unveiling a richer landscape of macrophage subsets and dynamic transitions between these functional states (Jackson et al., 2025). This nuanced understanding is critical, as the balance and timing of macrophage polarization are pivotal in determining the outcome of tissue response to injury or biomaterial implantation, steering the process towards successful regeneration or, conversely, towards chronic inflammation and fibrosis (Figure 4).

### 5.1 The M1/M2 paradigm in tissue response

#### 5.1.1 Pro-inflammatory phase (M1-dominant)

Typically induced by signals such as interferon-gamma (IFN-γ), lipopolysaccharide (LPS), or tumor necrosis factor-alpha (TNF-α), M1-like macrophages are crucial for the initial phase of tissue response. Their primary functions include host defense against pathogens, efficient clearance of cellular debris and apoptotic cells, and the recruitment of other immune cells to the site of injury or implantation. M1 macrophages achieve this by producing a range of pro-inflammatory cytokines (e.g., TNF-α, IL-1β, IL-6), reactive oxygen species (ROS), and inducible nitric oxide synthase (iNOS). While essential for initiating the healing process, a sustained or overly exuberant M1-dominant microenvironment can lead to excessive tissue damage, chronic inflammation, and ultimately, the development of pathological fibrosis.

#### 5.1.2 Repair phase (M2-dominant)

The transition to an M2-dominant phenotype is generally promoted by cytokines such as IL-4, IL-13, IL-10, and transforming growth factor-beta (TGF-β). M2-like macrophages play a critical role in the resolution of inflammation and the promotion of tissue repair and regeneration. Their functions include stimulating angiogenesis through the secretion of factors like vascular endothelial growth factor (VEGF) and fibroblast growth factor (FGF), promoting the deposition and remodeling of the extracellular matrix (ECM), and activating fibroblasts for organized collagen synthesis. M2 macrophages are characterized by the secretion of anti-inflammatory cytokines (e.g., IL-10, TGF-β) and the expression of specific markers such as CD206 (mannose receptor) and Arginase-1. A timely and effective switch from an M1- to an M2-dominant microenvironment is paramount for achieving successful tissue regeneration and preventing the onset of fibrotic scarring. The emerging complexity revealed by scRNA-seq suggests that future biomaterials may need to induce specific M2 subsets (e.g., M2a, M2c) tailored to the regenerative need, as not all “M2-like” states are uniformly beneficial; some may even contribute to fibrosis in certain contexts (Jackson et al., 2025).

### 5.2 Material-mediated macrophage polarization

Smart biomaterials offer powerful tools to actively guide macrophage polarization. This can be achieved by meticulously designing their intrinsic physicochemical properties, as detailed in Section 3, or by serving as vehicles for the controlled delivery of specific immunomodulatory molecules directly to the target microenvironment.

#### 5.2.1 Delivery of microRNAs (miRNAs)

MicroRNAs are small non-coding RNAs that act as crucial endogenous regulators of gene expression, capable of fine-tuning complex cellular processes, including macrophage activation and polarization (Saadh et al., 2025). The delivery of specific miRNAs, often encapsulated within extracellular vesicles (EVs) or other nanocarriers, represents a highly biomimetic and potentially potent strategy for immunomodulation. For instance, EVs loaded with miRNA-223 have been demonstrated to suppress the expression of M1-associated markers (such as iNOS, potentially by targeting transcription factor Pknox1 or modulating Notch/NLRP3 signaling pathways) and to promote M2 polarization. Specifically, exosome-delivered miR-223-3p has been shown to be enriched in M2-polarized microglia (the brain’s resident macrophages) and can suppress NLRP3-mediated pyroptosis in adjacent neurons, highlighting a neuroprotective role (Yang Q. et al., 2025). This approach leverages natural intercellular communication mechanisms and intracellular regulatory pathways, potentially leading to more stable and nuanced phenotypic shifts compared to stimulation with exogenous factors.

#### 5.2.2 Delivery of cytokines

Biomaterials can also be engineered for the sustained and localized release of cytokines known to directly influence macrophage polarization. For example, microspheres or hydrogels loaded with IL-4 have been effectively used to induce an M2 phenotype. Studies have shown that IL-4 released from calcium-enriched gellan gum (Ca-GG) hydrogel beads can promote M2 macrophage polarization, increase the expression of TGF-β1, and consequently enhance the osteogenic differentiation of bone mesenchymal stem cells (BMSCs) via the JAK1/STAT6 pathway (He et al., 2020).

#### 5.2.3 Delivery of other bioactive molecules

Beyond miRNAs and canonical cytokines, other bioactive molecules can be incorporated into biomaterials to steer macrophage behavior. For example, scaffolds designed to release ursolic acid, a natural compound with anti-inflammatory properties, have been shown to induce M2 macrophage polarization and improve outcomes in bone regeneration models (Jackson et al., 2025).

### 5.3 Dynamic regulation for optimal outcomes

The ideal immunomodulatory strategy rarely involves a simple, static shift towards one macrophage phenotype. Instead, optimal tissue regeneration often necessitates a dynamic regulation of macrophage polarization over time. This might entail an initial, controlled M1 phase to effectively clear debris and pathogens and to initiate the healing cascade, followed by a robust and sustained M2 phase to resolve inflammation, promote angiogenesis, and facilitate constructive tissue remodeling. A biomaterial that exclusively promotes M2 polarization from the outset might inadvertently impair the crucial initial inflammatory cleanup. Conversely, a material that indefinitely sustains M1 activity will likely lead to chronic inflammation and deleterious fibrosis. Smart biomaterials, with their inherent responsiveness and sophisticated controlled release capabilities, are uniquely positioned to orchestrate this complex temporal switching of macrophage phenotypes. By delivering the right signals at the right time, they can guide the microenvironment away from destructive fibrotic pathways and towards functional tissue restoration. This underscores the critical need for biomaterials that can achieve not just a specific immune state, but a programmed sequence of immune states, aligning with the natural choreography of healing.

## 6 Application cases of responsive biomaterials

The true potential of smart biomaterials is realized when their stimulus-responsive characteristics are precisely harnessed to address specific therapeutic challenges. This section highlights concrete examples where the dynamic nature of these materials is leveraged to modulate immune responses and promote regeneration or treat disease. A recurring theme in these applications is the strategic alignment of the material’s responsiveness with the unique pathological cues or physiological requirements of the target tissue or disease state. The success of these systems often depends on the specificity of this stimulus-response coupling; the chosen stimulus must be a reliable indicator of the target pathology, and the material’s response must be appropriately calibrated to the intensity and duration of that stimulus.

### 6.1 pH-responsive systems in cancer immunotherapy

The tumor microenvironment (TME) frequently exhibits a lower pH (typically pH 6.5–6.9) compared to normal physiological tissues (pH ∼ 7.4). This acidity arises from altered cancer cell metabolism, often referred to as the Warburg effect (increased glycolysis even in the presence of oxygen), and from poor vascular perfusion within the tumor mass (Karimi et al., 2025). This acidic TME not only supports tumor progression but can also actively suppress anti-tumor immune responses. pH-responsive nanoparticles are ingeniously designed to exploit this characteristic. These nanoparticles can preferentially accumulate in tumors, often aided by the enhanced permeability and retention (EPR) effect or through active targeting strategies. Once in the acidic TME, they are triggered to release their immunomodulatory payload.

The cargo can include immune checkpoint inhibitors (ICIs) such as anti-PD-L1 or anti-PD-1 antibodies, or immunostimulatory cytokines like IL-12. For example, nanoparticles have been developed to release anti-PD-L1 antibodies within the acidic TME, aiming to block the PD-1/PD-L1 immunosuppressive axis locally (Ge et al., 2025). Similarly, pH-responsive nano-vaccines, co-delivering antigens and adjuvants (like STING agonists), can show enhanced release and activity in acidic conditions, thereby promoting dendritic cell activation and priming robust anti-tumor T-cell responses, which can be further potentiated by systemic ICI therapy (Wang et al., 2025a). Some pH-responsive nanoparticles are even designed to neutralize the acidic TME by consuming H+ ions, which can itself have anti-tumor effects and improve the efficacy of other therapies (Liu et al., 2025). The localized release of these potent immunotherapeutics enhances their concentration at the tumor site, thereby boosting anti-tumor immunity while significantly reducing the systemic toxicities often associated with conventional, broadly acting immunotherapies.

### 6.2 Thermosensitive hydrogels for myocardial regeneration

Following a myocardial infarction (MI), the ischemic heart muscle undergoes a series of detrimental changes, including inflammation, cardiomyocyte death, and adverse remodeling leading to scar formation and impaired cardiac function. Regenerative strategies aim to promote angiogenesis (new blood vessel formation), reduce inflammation, and limit scarring to preserve heart function. Injectable thermosensitive hydrogels offer a promising platform for achieving these goals.

These materials are typically administered as a liquid solution directly into or onto the infarcted myocardial tissue. Upon reaching body temperature, they undergo a sol-gel transition, forming a gel *in situ* that can provide mechanical support to the weakened ventricular wall and serve as a localized reservoir for therapeutic agents (Yu, 2023).

A key application is the delivery of pro-angiogenic growth factors, such as Vascular Endothelial Growth Factor (VEGF). For instance, PEG-fibrinogen hydrogels loaded with VEGF have been shown to improve cardiac function, reduce scar size, and enhance endothelial cell motility and neovascularization in rodent MI models. The thermosensitive nature of the hydrogel ensures that it remains at the site of injury, providing sustained release of VEGF directly into the ischemic tissue. This localized delivery promotes the formation of new blood vessels, improves perfusion, and supports the survival of remaining cardiomyocytes, ultimately contributing to better functional recovery. The stimulus here is the physiological body temperature, which triggers the gelation and subsequent controlled release.

### 6.3 Enzyme-responsive scaffolds for scar inhibition

Pathological scarring, such as hypertrophic scars or keloids, results from dysregulated wound healing processes, often characterized by excessive deposition of disorganized extracellular matrix (ECM) and persistent inflammation. Matrix metalloproteinases (MMPs) are a family of enzymes that play a crucial role in ECM remodeling. While necessary for normal wound healing, their aberrant activity (either excessive or insufficient) can contribute to scar formation (Pakyari et al., 2013). Transforming growth factor-beta (TGF-β) isoforms also play complex roles: TGF-β1 and TGF-β2 are generally considered pro-fibrotic, promoting collagen synthesis and scar formation, whereas TGF-β3 has been suggested to have anti-fibrotic properties, potentially by promoting a more organized ECM structure or by increasing MMP activity for better remodeling.

Enzyme-responsive scaffolds can be designed to release therapeutic agents specifically in areas where target enzymes, like MMPs, are overexpressed. For scar inhibition, a scaffold could be engineered to incorporate TGF-β3 (or an inhibitor of TGF-β1/β2) linked via an MMP-cleavable peptide. In regions of high MMP activity, indicative of active and potentially aberrant ECM remodeling, the scaffold would be degraded by MMPs, leading to the localized release of TGF-β3. The released TGF-β3 could then act to counteract pro-fibrotic signals, promote a more balanced ECM deposition, and modulate fibroblast behavior, thereby reducing scar formation and encouraging a more regenerative healing outcome. For example, exogenous TGF-β3 has been noted to reduce collagen type I deposition by promoting its degradation via MMP-9. This strategy aims to leverage the pathological cue (MMP overexpression) to trigger a corrective therapeutic response.

### 6.4 Other responsive systems

Beyond pH, temperature, and enzymes, other stimuli are also being exploited. ROS-responsive biomaterials, for instance, can release therapeutic agents in environments with high oxidative stress, such as in cardiovascular diseases like atherosclerosis, or at sites of inflammation (Yuan et al., 2025). Light-responsive systems offer external spatiotemporal control over drug release or material properties, potentially useful in dermatological applications or superficial tissue engineering (Gheorghiu et al., 2021). Magnetic field-responsive materials can be guided to specific locations or triggered to release drugs using external magnetic fields, finding applications in targeted drug delivery and hyperthermia. These examples underscore the versatility of responsive biomaterials in tailoring therapeutic interventions to a wide array of challenging medical conditions by precisely matching material responsiveness to the specific disease context, often aiming to counteract pathological deviations and restore a more homeostatic microenvironment.

## 7 Biomimetic topology design: from cell migration to immune regulation

The native extracellular matrix (ECM) is far more than an inert scaffold, it is a dynamic and intricate network of macromolecules that provides essential structural support, mechanical integrity, and a rich tapestry of biochemical and topographical cues. These cues collectively orchestrate a wide range of cellular behaviors, including adhesion, migration, proliferation, differentiation, and intercellular communication.^18^ Biomimetic design in biomaterials science endeavors to replicate these critical ECM features in synthetic or engineered constructs. The goal is to create more physiologically relevant microenvironments that can actively instruct cells, including immune cells and stem cells, towards desired regenerative or immunomodulatory pathways (Wan et al., 2025). This approach signifies a shift from merely ensuring biocompatibility to actively *instructing* cellular behavior by recreating the instructive natural microenvironments.

### 7.1 ECM-mimetic surfaces and nanoscale fiber topology

A key strategy in biomimetic design involves engineering material surfaces with specific nanoscale topographical features—such as precisely dimensioned fibers, grooves, pores, or pillars—that mimic the architecture of natural ECM components like collagen fibrils. Fiber diameters typically explored are in the range of 50–500 nm, with some studies focusing on the 50–200 nm range as particularly relevant for mimicking native ECM structures (Liu et al., 2023).

Such nanoscale topography can exert a profound influence on immune cells, particularly macrophages. It can affect their adhesion, morphology (e.g., promoting elongation when cultured on aligned fibers or grooves), and polarization state. An elongated macrophage morphology is often correlated with an M2-like (anti-inflammatory and pro-reparative) phenotype. Specific nanotopographies have been shown to promote the secretion of anti-inflammatory cytokines, such as IL-10, while concurrently downregulating the production of pro-inflammatory mediators. For example, biocompatible Mg-Al layered double hydroxide (LDH) nano-sheet arrays constructed on titanium surfaces significantly promoted the polarization of macrophages towards the M2 phenotype, an effect associated with the activation of the PI3K-AKT-mTOR signaling pathway and increased gene expression of integrin β2 and focal adhesion kinase (FAK) (Zheng et al., 2022). Similarly, PCL, PDMS, or PLA surfaces with micro- or nanoscale grooves can induce macrophage elongation and bias their polarization towards an M2 state (Dutta et al., 2023). These cellular responses are mediated through interactions with cell surface receptors, notably integrins, which then trigger cytoskeletal rearrangements (involving actin filaments and microtubules) and activate downstream mechanotransduction pathways (Momotyuk et al., 2025).

Beyond immune cells, ECM-mimetic topographies are also powerful tools for guiding stem cell behavior. These physical cues can direct the differentiation of stem cells towards specific lineages, such as osteogenic, chondrogenic, or neurogenic fates, by providing a more physiologically relevant context (Yi et al., 2022). For instance, highly ordered TiO_2_ nanotube structures fabricated on Ti-6Al-4V alloy surfaces have been demonstrated to stimulate the osteogenic differentiation of mesenchymal stem cells (MSCs) (Shi et al., 2023). These topographies also influence stem cell adhesion, proliferation, and spatial organization.

### 7.2 3D-printed microenvironments

Additive manufacturing techniques, particularly 3D printing and bioprinting, have revolutionized the ability to fabricate scaffolds with precisely controlled three-dimensional architectures (Wan et al., 2025). This technology allows for the creation of biomimetic structures that replicate the hierarchical features of natural bone, including porous, layered, and gradient-distributed structures (Huang et al., 2025). Parameters such as porosity, pore size, interconnectivity, and even gradients of these features can be meticulously designed.

Scaffolds featuring gradient porosity are particularly interesting as they can mimic the structural heterogeneity of natural tissues, like bone, which transitions from a dense cortex to a porous trabecular interior. Optimized pore sizes (often cited in the 100–500 µm range) and high interconnectivity are crucial for facilitating the infiltration of immune cells deep into the scaffold structure. This allows for effective interaction between the cells and the material, enabling immune cells to participate actively in the wound healing and tissue integration process. Such porous networks are also vital for enhancing the diffusion of nutrients and oxygen to, and the removal of waste products from, cells residing within the scaffold, which is essential for maintaining cell viability and function throughout the construct and for promoting angiogenesis (Huang et al., 2025). The ability to create such gradient porosities using 3D printing is significant not only for nutrient diffusion but also for potentially establishing *functional zones* within a scaffold. These zones could guide a sequence of cellular events or create distinct niches for different cell types, thereby mimicking the complex spatial and temporal organization of natural tissue development and immune responses.

By fostering favorable immune cell infiltration (e.g., encouraging M2 macrophage recruitment), promoting robust vascularization, and facilitating seamless tissue integration, well-designed 3D printed scaffolds can significantly reduce the likelihood of adverse foreign body responses and the formation of fibrotic capsules that would otherwise impede implant function. For example, biomimetic ECM nerve guidance conduits (NGCs) with 3D interconnected porous networks have been shown to support peripheral nerve regeneration and promote M2 macrophage polarization (Wan et al., 2025).

### 7.3 Nuclear mechanosensing and cell migration

Cells possess sophisticated mechanisms to sense mechanical cues from their substrate, and these signals are intricately transmitted through the cytoskeleton to the nucleus. The nucleus itself is not a passive organelle; it can deform in response to these forces, and this “nuclear mechanosensing” plays a pivotal role in regulating gene expression, cell fate decisions, and cell migration (Yi et al., 2022). Substrate stiffness, for example, can influence cell migration speed and direction, a phenomenon known as durotaxis, where cells preferentially migrate towards stiffer regions (Van Helvert et al., 2018).

Forces are transmitted to the nucleus via structures like the LINC (Linkers of the Nucleoskeleton and Cytoskeleton) complex and nuclear lamins. These transmitted forces can lead to alterations in chromatin organization and, consequently, changes in gene transcription programs that govern migratory machinery and other cellular functions. Key mechanosensitive signaling pathways, such as YAP/TAZ, are also intimately involved. Nuclear deformation may be a critical, even rate-limiting, step for cells migrating through confined 3D environments, as the nucleus is typically the largest and stiffest organelle. This involvement of nuclear mechanosensing in response to substrate properties suggests that biomaterials can influence cell behavior at a very fundamental level—by directly impacting nuclear mechanics and gene regulation. This opens up exciting possibilities for influencing cell fate and function in ways that are not solely dependent on classical surface receptor-ligand interactions, potentially allowing for the “mechanical reprogramming” of cells.

Understanding how cells sense and respond to scaffold mechanics via these nuclear pathways offers novel insights for designing “pro-regenerative scaffolds”. Such scaffolds could incorporate defined stiffness gradients or specific topographical cues that leverage nuclear mechanosensing to actively guide cell migration (e.g., to recruit desired regenerative cell populations to an injury site) and to promote pro-regenerative cellular phenotypes.

In essence, topological engineering of biomaterials is about converting physical cues—the material’s architecture and mechanics—into specific biochemical and functional instructions for cells. This allows for a more subtle and potentially more effective form of “stealth” or “instructive” immune modulation, where the physical design of the material, rather than solely relying on released drugs, dictates the immune outcome and directs tissue regeneration.

## 8 Multiscale modeling: from molecular mechanisms to clinical prediction

**FIGURE 5:**
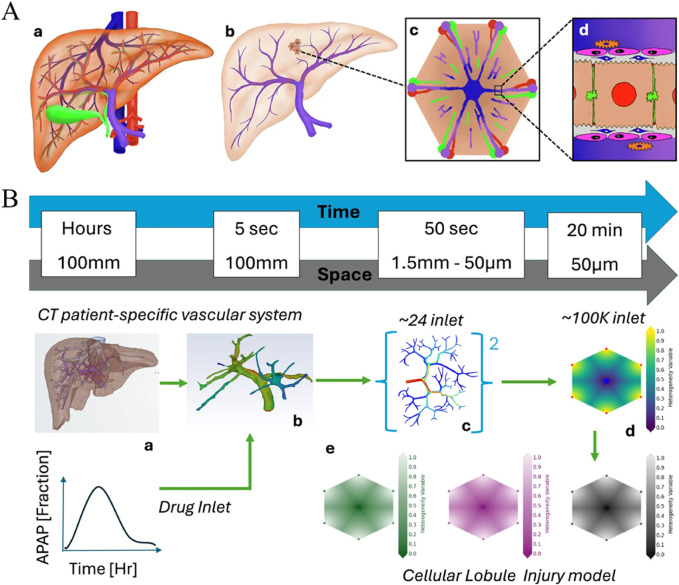
Illustration depicting a two-part diagram of liver vascular modeling. **(A)** shows a liver anatomy model progressing from a full liver **(A-a)** to a detailed view at the cellular level **(A-d)**. **(B)** outlines a timeline and spatial scale from CT patient-specific vascular imaging **(B-a)** through various drug inlets **(B-b, B-c)** to a cellular lobule model **(B-d)**, showing drug distribution over time. Graphs and gradient color scales indicate drug concentration and distribution, illustrating acetaminophen (APAP) fraction and injury models.

The design and optimization of smart immunomodulatory biomaterials represent a formidable challenge due to the vast and intricate design space. This space encompasses numerous variables, including material composition, diverse physicochemical properties (stiffness, topography, charge, wettability), complex drug release kinetics, and the multifaceted biological interactions that ensue upon implantation. Traditional experimental approaches, which often rely on iterative trial-and-error, are inherently time-consuming, resource-intensive, and may not always provide a deep mechanistic understanding of the observed phenomena. Computational modeling, spanning multiple scales from molecular interactions to tissue-level responses, offers a powerful and increasingly indispensable toolkit to complement and guide experimental efforts, thereby accelerating the design-build-test-learn cycle in biomaterials science (Chen et al., 2025; Malka-Markovitz et al., 2025). This progression from molecular dynamics (MD) simulations to agent-based models (ABMs) and then to organ-on-chip (OoC) platforms represents a hierarchical strategy for tackling complexity, systematically bridging the gap from fundamental molecular interactions to cellular behaviors and, ultimately, to tissue and organ-level responses (Figure 5).

### 8.1 Molecular dynamics (MD) simulations

MD simulations are computational techniques that track the physical movements of atoms and molecules over time, governed by the laws of physics. They can provide detailed, atomistic or coarse-grained insights into molecular interactions at interfaces, which is particularly relevant for understanding the initial events that occur when a biomaterial is introduced into a biological environment (Rahmani et al., 2025).

Upon implantation, the very first interaction is the adsorption of host proteins onto the biomaterial surface, forming a “conditioning film”. This protein layer critically mediates subsequent cellular interactions and dictates the nature of the ensuing immune response. MD simulations can predict how different plasma proteins (e.g., albumin, fibrinogen, immunoglobulins, vitronectin) interact with various material surfaces. These simulations can elucidate adsorption affinities, predict conformational changes that proteins may undergo upon surface binding (which can expose new epitopes or alter protein function), and help to understand the competitive adsorption processes that determine the final composition of the adsorbed layer (the Vroman effect) (Brash et al., 2019).

By understanding and predicting these protein adsorption phenomena, MD simulations contribute significantly to optimizing the immune compatibility of biomaterials. For instance, simulations can guide the design of surfaces that minimize non-specific protein adsorption, a strategy often employed to reduce the foreign body response and improve biocompatibility (e.g., through PEGylation) (Rahmani et al., 2025). Conversely, MD can aid in engineering surfaces that selectively bind specific proteins known to promote favorable immune responses, such as anti-inflammatory or pro-regenerative molecules.

### 8.2 Agent-based models (ABM)

ABM is a computational modeling approach that simulates the actions and interactions of autonomous “agents”—which can represent individual cells (like macrophages or fibroblasts), molecules, or even larger entities—within a defined environment. Each agent follows a set of rules governing its behavior and interactions, and the simulation tracks how these individual actions lead to emergent, system-level behaviors (Brown et al., 2011).

In the context of immunomodulatory biomaterials, ABMs are powerful tools for simulating macrophage-material interactions. Agents representing macrophages can be programmed with rules for adhesion to the biomaterial surface, migration patterns influenced by material topography or chemotactic gradients, phagocytic activity, and cytokine secretion profiles that are dependent on the material’s properties (e.g., stiffness, surface chemistry) and the local concentration of signaling molecules. By defining rules for M1 and M2 polarization based on these perceived stimuli (e.g., material cues, paracrine signals from other cells), ABMs can forecast the temporal and spatial dynamics of macrophage phenotypes within the microenvironment surrounding the implant.

Crucially, ABMs can integrate the behavior of other relevant cell types, such as fibroblasts, which are key players in ECM production and fibrosis. By modeling fibroblast proliferation, migration, and collagen deposition in response to macrophage-derived signals (like TGF-β) and material characteristics, these models can predict the formation of granulation tissue and assess the risk of developing a fibrotic capsule around the implant. For example, an ABM investigating lung fibrosis identified M2 macrophages as primary contributors to increased collagen deposition, driven by TGF-β dynamics (Islam et al., 2023). The predictive capability of ABMs for fibrosis risk signifies a critical step towards *in silico* safety and efficacy assessment, potentially reducing reliance on animal testing and accelerating the development of safer biomaterials.

### 8.3 Preclinical validation platforms: Organ-on-chip (OoC) systems

OoC systems are advanced *in vitro* models that utilize microfluidic technology to create microscale devices lined with living cells, aiming to replicate the key structural, functional, and physiological complexity of human tissues and organs. These platforms allow for the co-culture of multiple cell types, the incorporation of ECM components, and the application of physiologically relevant mechanical cues, such as fluid shear stress (mimicking blood or interstitial flow) and cyclic strain (mimicking breathing or muscle contraction).

OoC systems are increasingly used as preclinical validation platforms for evaluating the immunomodulatory efficacy of biomaterials under conditions that more closely mimic the *in vivo* setting than traditional static 2D cell cultures. “Immune-organ-on-a-chip” models can be developed to study the interaction of biomaterials with immune cells (e.g., macrophages, T cells, neutrophils) in a dynamic and tissue-specific context (Mattei et al., 2021). Biomaterial samples or coatings can be integrated into the microfluidic channels, and their influence on immune cell adhesion, migration, activation, polarization, and cytokine production can be monitored in real-time, often under controlled material mechanics and flow conditions. The integration of material mechanics and fluid shear stress in OoC systems is particularly vital because immune responses *in vivo* are profoundly influenced by the dynamic mechanical environment of tissues. Ignoring these factors can lead to *in vitro* results that do not accurately predict *in vivo* performance. Thus, OoCs provide a more stringent and relevant testing ground for immunomodulatory biomaterials.

### 8.4 Computational-experimental synergy

The true power of these multiscale modeling approaches is realized through their synergistic integration with experimental validation. Computational models can guide experimental design by identifying critical parameters, generating testable hypotheses, or predicting potential outcomes, thereby making experimental work more focused and efficient. Conversely, experimental data, including that derived from OoC platforms and, ultimately, well-designed animal models, are essential for calibrating model parameters, validating model predictions, and refining the underlying assumptions of the models. This iterative loop of computational prediction and experimental verification accelerates the rational design process, facilitates a deeper understanding of complex biomaterial-host interactions, and ultimately shortens the translational pipeline for novel smart immunomodulatory biomaterials.

## 9 Clinical translation status and challenges

Despite the remarkable pace of innovation and the compelling preclinical evidence for smart immunomodulatory biomaterials, their journey from laboratory discovery to routine clinical use is fraught with significant hurdles. The landscape of clinically approved or advanced-stage investigational products in this specific niche remains relatively sparse, underscoring a substantial “valley of death” in translation. This gap suggests that current preclinical models and evaluation criteria may not be sufficiently predictive of human responses, or that the inherent complexity and novelty of these systems introduce unforeseen challenges during human trials and manufacturing scale-up.

### 9.1 Current landscape of clinically investigated/approved products

The direct translation of smart biomaterials specifically designed for active immune modulation is an emerging area, with few products having reached full market approval based explicitly on such mechanisms.

**Silk fibroin nerve conduits**: Silk fibroin has garnered attention for nerve regeneration due to its biocompatibility and tunable properties. Preclinical studies suggest that silk fibroin conduits (SFCs) can promote a favorable immune microenvironment, including the recruitment of M2 macrophages, which is thought to contribute to nerve repair (Matsuo et al., 2025). The user query indicated that SFCs have completed Phase I trials for this indication. However, a closer examination of available literature reveals some ambiguity. While pilot studies, such as one involving the “SILKBridge” conduit for digital nerve reconstruction in a small number of patients (four individuals), have reported promising safety and efficacy outcomes including good biointegration and sensory recovery (Matsuo et al., 2025), these may not constitute formal, large-scale Phase I trials focused specifically on macrophage polarization as a primary endpoint. Some reviews even suggest that current evidence does not definitively support the superiority of existing nerve repair devices over standard surgical techniques and note potential adverse events. This highlights the complexities of early clinical translation and the need for robust, well-controlled trials, explicitly states that “no SFC has been applied in clinical practice worldwide”, suggesting that while research is active, widespread clinical adoption based on immunomodulatory claims is not yet a reality (Wang et al., 2018; Politikou et al., 2025).

### 9.2 Thermosensitive hydrogels with IL-10 for diabetic ulcer repair

The concept of using thermosensitive hydrogels to deliver anti-inflammatory cytokines like IL-10 to chronic wounds, such as diabetic foot ulcers (DFUs), is therapeutically sound. IL-10 is a potent anti-inflammatory cytokine that can promote M2 macrophage polarization and aid in resolving chronic inflammation characteristic of DFUs. The user query suggested such systems are in Phase II trials. However, specific PubMed-indexed evidence from the provided research for this exact formulation (thermosensitive hydrogel + IL-10) in Phase II trials is not readily apparent. Meta-analyses confirm that hydrogel dressings, in general, are effective in improving healing rates for DFUs compared to conventional dressings (Zhao et al., 2024a). Some Phase II trials have investigated hydrogel sheets containing human adipose-derived mesenchymal stem cells (hAT-MSCs) for DFU treatment, reporting good safety and improved wound closure (Sierra-Sánchez et al., 2021), but these do not specifically involve IL-10 or thermosensitivity as the primary smart feature for IL-10 release. While thermosensitive hydrogels carrying other growth factors (e.g., KGF-2, FGF-21) have shown promise in preclinical diabetic wound models (Yang et al., 2020), and the general potential of hydrogels for DFU is recognized (Nifontova et al., 2024), a clear trail for an IL-10-loaded thermosensitive hydrogel in Phase II trials is not strongly supported by the provided snippets.

It is important to note that the broader field of immunomodulatory therapeutics, including antibodies, cell-based therapies, and viral therapies, has seen variable success in treating cancers and autoimmune diseases, providing a relevant context for the challenges faced by biomaterial-based approaches (Nash et al., 2021).

### 9.3 Key translation bottlenecks and challenges

#### 9.3.1 Long-term biosafety

Ensuring the long-term safety of smart biomaterials within the human body is a paramount concern. This encompasses potential chronic inflammation, unforeseen immunogenicity of the material itself or its degradation byproducts, the risk of eliciting unintended systemic immune effects, and general toxicity. Degradation products must be non-toxic and efficiently metabolized or cleared by the body; their accumulation or unexpected chemical interactions can lead to adverse local or systemic reactions. For materials designed for dynamic responsiveness, the long-term stability and predictability of this response *in vivo*, over months or years, remain a significant area of investigation and concern (Ullah et al., 2025).

#### 9.3.2 Scalable and reproducible manufacturing

The transition from lab-scale synthesis of often complex smart biomaterial formulations to large-scale, cost-effective, and Good Manufacturing Practice (GMP)-compliant manufacturing processes is a major engineering and logistical hurdle. Maintaining stringent batch-to-batch consistency in terms of material physicochemical properties, drug loading efficiency, release kinetics, and stimulus-responsiveness is critical for regulatory approval and reliable clinical performance. This is particularly challenging for materials with intricate nano- or micro-architectures, multi-component systems like engineered EVs, or those requiring precise surface modifications. The cost-effectiveness of these advanced manufacturing processes is also a crucial determinant for commercial viability and accessibility.

#### 9.3.3 Reliability of dynamic response *in vivo*

Smart biomaterials are predicated on their ability to respond to specific physiological cues. However, the *in vivo* microenvironment is vastly more complex, dynamic, and variable than controlled *in vitro* conditions. Ensuring that the material reliably senses the intended stimulus (e.g., a specific pH change, enzyme level, or temperature) and elicits the correct therapeutic response in the presence of numerous other fluctuating biological factors (e.g., diverse cell populations, varying protein concentrations, altered metabolic states) is a profound challenge. The immune system itself is highly dynamic and adaptive; a material’s response could potentially be altered by the very immune cells it aims to modulate, leading to unpredictable feedback loops or a gradual loss of efficacy over time. Phenomena such as protein biofouling on the material surface, cellular encapsulation, or unexpected biochemical interactions can dampen, block, or otherwise alter the material’s intended responsiveness.

#### 9.3.4 Predictive preclinical models

A persistent challenge in biomaterial translation is the often-poor correlation between results from standard *in vitro* assays or conventional animal models and actual human clinical outcomes, particularly concerning immune responses. There is an urgent need for more sophisticated and human-relevant preclinical models, such as humanized mouse models, patient-derived organoids, or advanced OoC systems incorporating human cells and physiological cues, to better evaluate immunomodulatory efficacy and predict safety profiles before embarking on costly human trials.

#### 9.3.5 Regulatory pathways

The regulatory landscape for smart biomaterials, especially those exhibiting dynamic, responsive, or autonomous functions, is continually evolving. These products frequently blur the lines between traditional categories of medical devices and pharmaceutical drugs, often being classified as combination products. This necessitates navigating complex and sometimes less-defined regulatory pathways. The development of standardized testing protocols, clear guidance documents from regulatory agencies, and consensus on appropriate endpoints for these novel therapies are crucial for streamlining their path to clinical approval.

### 9.4 The role of interdisciplinary collaboration and regulatory innovation

Successfully overcoming these multifaceted translational hurdles mandates intensive and sustained collaboration among experts from diverse fields, including materials scientists, immunologists, bioengineers, pharmacologists, clinicians, and regulatory affairs specialists. A synergistic approach is essential to bridge the gap between fundamental discovery and clinical application. Furthermore, innovation in regulatory science itself is critical. This includes the development of new assessment frameworks, adaptive trial designs, and guidelines that are specifically tailored to the unique characteristics and complexities of smart, responsive, and immunomodulatory biomaterials. A more adaptive and collaborative regulatory environment will be key to fostering the safe and timely clinical adoption of these transformative technologies (Nash et al., 2021). The complexity of these materials may ultimately demand entirely new paradigms for quality control, risk assessment, and post-market surveillance. Table 3 provides an illustrative overview of some clinically investigated smart immunomodulatory biomaterial concepts, based on the user query and the information available within the provided research snippets.

**TABLE 3:** Overview of clinically investigated smart immunomodulatory biomaterials (illustrative).

Biomaterial system/Product concept	Target application	Primary immunomodulatory strategy	Reported clinical trial phase (PubMed-cited evidence from snippets)	Key reported outcomes/Challenges (PubMed-cited from snippets)
Silk Fibroin Nerve Conduits (e.g., SILKBridge)	Peripheral Nerve Regeneration	(a) Promotion of M2 macrophage polarization. (b) Provision of regenerative microenvironment.	Preclinical evidence for M2 polarization. Pilot human study (n=4, “SILKBridge”) reported safety and sensory recovery (Thomson et al., 2022). Formal Phase I focused on immunomodulation not clearly detailed.	Outcomes: Good biointegration, satisfactory sensory recovery in pilot study. Challenges: Ambiguity regarding formal Phase I trials specifically assessing immunomodulation. Some reviews question superiority over standard repair and note potential for adverse events. Need for larger, controlled trials. “No SFC has been applied in clinical practice worldwide”.
Thermosensitive Hydrogel + IL-10	Diabetic Ulcer Repair	(a) Localized delivery of anti-inflammatory cytokine IL-10. (b) Promotion of M2 macrophage polarization. (c) resolution of chronic inflammation.	Phase II suggested by user query, but specific evidence for this exact formulation (thermosensitive + IL-10) in Phase II is not strongly supported by provided snippets (Zhao et al., 2024a).	Outcomes (General Hydrogels for DFU): Improved healing rate, shortened healing time. hMSC-loaded hydrogels show promise in Phase II. Challenges: Lack of specific Phase II trial data for IL-10-loaded thermosensitive hydrogels in the provided snippets. Need for robust clinical validation of this specific combination.

## 10 Future perspectives

The field of smart biomaterials as active immune modulators is on a trajectory of rapid advancement, poised to transition from materials that simply respond to their environment to those that intelligently and dynamically engineer it. Future innovations are expected to focus on enhancing the precision, personalization, and potency of these immunomodulatory interventions, heralding an era of “precision immune engineering”.

### 10.1 Towards truly dynamic material systems

The next frontier in smart biomaterials involves the development of systems capable of truly dynamic, real-time interaction with and control over the biological microenvironment.

**Integration of optogenetics and self-feedback loops:** A particularly exciting avenue is the integration of optogenetic tools with biomaterials (Gheorghiu et al., 2021). Optogenetics utilizes light-sensitive proteins to control cellular processes (e.g., gene expression, ion channel activity, cell signaling) with high spatiotemporal precision using external light as a non-invasive trigger. Incorporating optogenetic elements into biomaterials or using them to control cells interacting with biomaterials could allow for on-demand, reversible, and finely tuned modulation of the material’s properties or the release of therapeutic agents in real-time. This could enable clinicians to dynamically adjust therapy based on patient response. Furthermore, the development of materials with intrinsic self-feedback loops represents a significant step towards autonomous function (Narkar et al., 2022). Such systems would integrate biosensors to detect changes in the local microenvironment (e.g., levels of specific biomarkers, pH shifts, temperature fluctuations) and autonomously adjust their behavior—such as altering drug release rates or modifying their physical properties—in a closed-loop fashion. This aligns with the concept of truly autonomous materials that can maintain local homeostasis or optimize therapeutic effects without continuous external intervention, mimicking the sophisticated feedback control mechanisms inherent in biological systems. The emerging domain of “metabolic cybergenetics”, which uses computer interfaces for real-time feedback control over biological processes, could also be integrated with responsive biomaterials (Gheorghiu et al., 2021).

### 10.2 Personalized design strategies

The heterogeneity of human immune responses and disease pathologies necessitates a shift away from “one-size-fits-all” biomaterial solutions towards personalized approaches.

**Tailoring to patient-specific immune profiles:** Future biomaterial design will likely involve tailoring material characteristics—such as stiffness, surface topography, degradation kinetics—and the profile of released immunomodulatory factors based on an individual patient’s unique immune status, genetic predispositions, specific disease phenotype, or even their microbiome. This could involve pre-implantation screening of patients for relevant immune biomarkers to predict their likely response to a particular biomaterial or to guide the selection or custom design of the most appropriate immunomodulatory strategy. The integration of multi-omics data—including genomics, transcriptomics, proteomics, and metabolomics—will be crucial for developing these patient-specific profiles and informing biomaterial design (Chen et al., 2025). For instance, single-cell RNA sequencing (scRNA-seq) could guide the strategic incorporation of growth factors or cytokines into biomaterial platforms based on the patient’s cellular landscape.

**AI-driven biomaterial design:** Artificial intelligence (AI) and machine learning (ML) algorithms are set to revolutionize this personalization effort. AI/ML can analyze vast datasets comprising material properties, cellular responses *in vitro* and *in vivo*, and patient clinical data to identify complex patterns and predict optimal biomaterial designs tailored to individual patient needs or specific therapeutic goals. This computational power will significantly accelerate the development and optimization of personalized immunomodulatory therapies.

### 10.3 Multimodal synergistic therapies

The efficacy of immunomodulation can often be significantly enhanced by combining different therapeutic modalities that act synergistically.

**Combining local and systemic immunomodulation:** A particularly promising strategy, especially in cancer therapy, involves combining localized, biomaterial-mediated immunomodulation with systemic treatments like immune checkpoint blockade (ICB). Smart biomaterials can be employed to deliver immunostimulatory agents (e.g., STING agonists, TLR agonists, cytokines, or even oncolytic viruses) directly to the tumor microenvironment. This localized action can help to create a pro-inflammatory milieu, enhance antigen presentation, increase T-cell infiltration, and thereby sensitize immunologically “cold” tumors to the effects of systemic ICIs, effectively converting them into “hot” tumors that are more responsive to checkpoint inhibitors. This approach aims to improve the efficacy of ICB while potentially reducing systemic side effects. Similarly, combining radiotherapy with ICB has shown synergistic anti-tumor effects, partly by inducing immunogenic cell death and favorably altering the TME; smart biomaterials could further amplify this synergy by delivering radiosensitizers, additional immunomodulators, or agents that mitigate radiation-induced toxicity to healthy tissues (Zhao et al., 2024b).

**Material-mediated synergies:** Biomaterials can also enhance multimodal therapies by protecting therapeutic agents from degradation, ensuring their sustained and localized co-delivery, and enabling the administration of combinations of drugs with different physicochemical properties or mechanisms of action that would be difficult to achieve effectively through systemic routes alone.

The convergence of these advanced material design principles, personalized approaches, and synergistic therapeutic strategies signifies the maturation of the field towards “precision immune engineering”. Smart biomaterials are no longer envisioned merely as tissue replacements or passive drug carriers. Instead, they are emerging as sophisticated tools capable of actively and precisely interacting with the host immune system, dynamically adapting to the biological microenvironment, and orchestrating complex cellular and molecular events to promote tissue regeneration, resolve inflammation, combat disease, and ultimately restore health. The potential lies not only in their ability to replace or repair tissues but in their capacity to reshape the very microenvironments that govern life and healing. While significant challenges in translation remain, the ongoing innovation in this interdisciplinary field promises a future where smart biomaterials play an increasingly integral role in personalized and highly effective medical interventions.

## 11 Conclusion

The field of smart biomaterials has rapidly evolved from passive scaffolds to dynamic platforms capable of actively modulating host immune responses, thereby shaping pro-regenerative microenvironments. This review has charted this evolution, highlighting the core innovations in dynamic responsiveness and active regulatory capabilities that define these advanced materials. The classification from inert to active, responsive, and ultimately autonomous systems underscores a clear trajectory towards increasing sophistication and biomimicry, with materials designed to sense and intelligently react to physiological and pathological cues such as pH, temperature, and enzymatic activity.

A central theme is the pivotal role of macrophage polarization in dictating the balance between tissue repair and fibrosis. Smart biomaterials offer unprecedented tools to influence this balance by precisely controlling their physicochemical properties—stiffness, topology, and degradation rate—which serve as potent, non-pharmacological regulators of macrophage phenotype. Furthermore, strategies for the spatiotemporal controlled release of immunomodulatory factors, including cytokines, small-molecule drugs, and engineered extracellular vesicles, allow for targeted interventions that maximize therapeutic efficacy while minimizing systemic toxicity. Application cases in cancer immunotherapy, myocardial regeneration, and scar inhibition demonstrate the tangible benefits of aligning material responsiveness with specific therapeutic needs.

The design of biomimetic topologies, mimicking the native ECM at the nanoscale and leveraging 3D printing for complex microenvironments, further refines the ability to convert physical cues into biochemical instructions for immune regulation and regenerative cell guidance. Concurrently, multiscale modeling approaches, from molecular dynamics simulations of protein adsorption to agent-based models of cell-material interactions and organ-on-chip validation platforms, are accelerating the rational design and preclinical assessment of these complex systems.

Despite these significant advances, the clinical translation of smart immunomodulatory biomaterials faces substantial hurdles. Long-term biosafety, the challenges of scalable and reproducible manufacturing, ensuring the reliability of dynamic responses *in vivo*, and navigating evolving regulatory pathways remain key bottlenecks. Overcoming these requires concerted interdisciplinary collaboration and continued innovation in both material science and regulatory frameworks.

Looking ahead, the future of smart biomaterials is exceptionally promising. The integration of technologies like optogenetics and self-feedback loops will enable real-time microenvironment control, pushing towards truly dynamic and autonomous material systems. Personalized design strategies, informed by patient-specific immune profiles and powered by AI-driven algorithms and multi-omics data, will tailor therapies to individual needs. Moreover, multimodal synergistic approaches, such as combining localized material-mediated immunomodulation with systemic treatments like immune checkpoint blockade, are poised to significantly enhance therapeutic efficacy, particularly in challenging diseases like cancer.

In conclusion, smart biomaterials are not merely replacing damaged tissues; they are actively engineering the biological milieu. By providing precise control over immune cell behavior and the local microenvironment, they are ushering in an era of “precision immune engineering”. The continued exploration and development of these sophisticated platforms hold the key to unlocking novel and highly effective treatments for a wide range of debilitating conditions, fundamentally transforming the landscape of regenerative medicine and therapeutic intervention.
